# Random lasing from structurally-modulated silk fibroin nanofibers

**DOI:** 10.1038/s41598-017-04881-5

**Published:** 2017-07-03

**Authors:** Soocheol Kim, SungYeun Yang, Seung Ho Choi, Young L. Kim, WonHyoung Ryu, Chulmin Joo

**Affiliations:** 1 School of Mechanical Engineering, Yonsei University, Seoul, 03722 Republic of Korea; 2 Weldon School of Biomedical Engineering, Purdue University, West Lafayette, Indiana, 47907 USA

## Abstract

Structural arrangement and dimension play vital roles in wave transport and amplification as they can restrict the volume explored by the waves. However, it is challenging to systematically investigate the interplay among structural, optical, and mechanical properties, in part because of limited experimental platforms that modulate the structural arrangement in a continuous manner. We present light amplification action in Rhodamine B doped silk fibroin (SF) nanofibrous scaffolds and its modulation via the control of the alignment or directionality of SF nanofibers through an electrospinning procedure. Random lasing features of such scaffolds are examined as a function of structural arrangement of the SF nanofibers, and optical-structural-mechanical relationships of the SF-based structures are examined. As SF nanofibers are aligned parallel undergoing a transition from three to quasi-two dimension, light amplification features (e.g., lasing threshold and output power) enhanced, which also strongly correlated with mechanical characteristics (i.e., Young’s moduli) of the scaffolds. We confirm such optical characteristics using quasi-mode analyses based on the finite element method. We further demonstrate non-contact, *in situ* measurement of alternations in lasing features of the scaffolds while the specimens are under tensile loads. These results may highlight potential utility of the scaffolds as a flexible and biocompatible sensor.

## Introduction

Amplified spontaneous emission or stimulated emission can be obtained through multiple light scattering by disordered scattering structures^1, 2^. Disordered arrangement of the cavity in a conventional laser has been thought as deteriorating the lasing performance, but it has been realized that multiple scattering from disordered structures can serve as feedback for light amplification and lasing. Since the theoretical studies^3, 4^ and experimental observations^5–8^, significant efforts have been focused on understanding the underlying physics of disordered cavities and implementing random lasing systems in many types of material systems^9–11^. Some interesting applications of random lasing have been reported, which include speckle-free optical imaging^12^, remote temperature sensing^13^, self-formed lasing from polymer-dispersed liquid crystal^14^, paper-based self-formed lasing^15^, and biosensors^16–18^.

One of the interesting avenues toward the realization of random lasers involves the implementation with biocompatible or biodegradable materials. Many random structures can be found in nature, which for example, provide mechanisms for body temperature regulation^19^, camouflage^20^, and structural coloring^21^. In these respects, researchers have measured light amplification effects in butterfly wings^22^, cicada wings^23^, human tissues^24^, and bone tissue^25^. Random lasing in biological tissue has also been exploited to detect cancerous tissue^24^ and monitor the changes in micro-structures under various mechanical conditions, observing that the optical characteristics are extremely sensitive to the micro- or nanoscale local perturbation of structures^26^.

Silk fibroin (SF), recently highlighted biocompatible material, has been extensively studied because of the tunability of its mechanical and optical properties. Diverse optical components such as waveguides^27^, microlens arrays^28^, and photonic crystals^29, 30^ have been fabricated, and lasing from SF nanograting structures^31^ has been demonstrated. Random lasing in SF-based photonic glasses has also been demonstrated, which are composed of bulk silk fibroin structured with pores of sizes comparable with the wavelength of light^32^. For applications in regenerative tissue engineering and drug delivery, SF has been structured in the forms of sutures, films^33, 34^, porous scaffolds^35, 36^, electrospun nanofibrous scaffolds^37, 38^, and micro- or nano-patterned structures^39, 40^.

Among these engineered structures, three-dimensional (3D) scaffolds composed of SF nanofibers are particularly attractive because of their large surface-to-volume ratio. These structures, for example, have been utilized as the scaffolds for cell proliferation and differentiation^37^, and its utility as artificial implants was also assessed. The measurements of the mechanical properties of such scaffolds revealed that the structural arrangement of the SF nanofibers greatly affected their mechanical properties^41^. On the other hand, random arrangement of SF nanofibers may provide a platform for light amplification. Incorporation of active dopants such as fluorescent dye molecules into the SF nanofibrous scaffolds could realize a unique photonic device that functions as a biocompatible scaffold, while providing optical signatures sensitive to local mechanical and structural features. The device is also flexible and readily adapted to biological host systems. Yet, its experimental demonstration and the relationship of its lasing characteristics with structural and mechanical properties have not been demonstrated, to our best knowledge.

Here, we demonstrate biocompatible random lasers based on SF nanofibers and the modulation of its lasing behaviors through the control of SF nanofiber arrangement. The measured lasing properties are compared against the structural and mechanical properties to understand the interplay among structural, optical, and mechanical properties in the scaffolds. The experimental results are further validated by numerical calculations of lasing modes with the finite element method (FEM). *In situ* measurement of changes of lasing features of the SF nanofibers is also presented, as the scaffolds are under tensile loads, suggesting potential application of the SF nanofibrous random lasers.

## Results

### Structurally-modulated SF nanofibrous scaffolds

**Figure 1:**
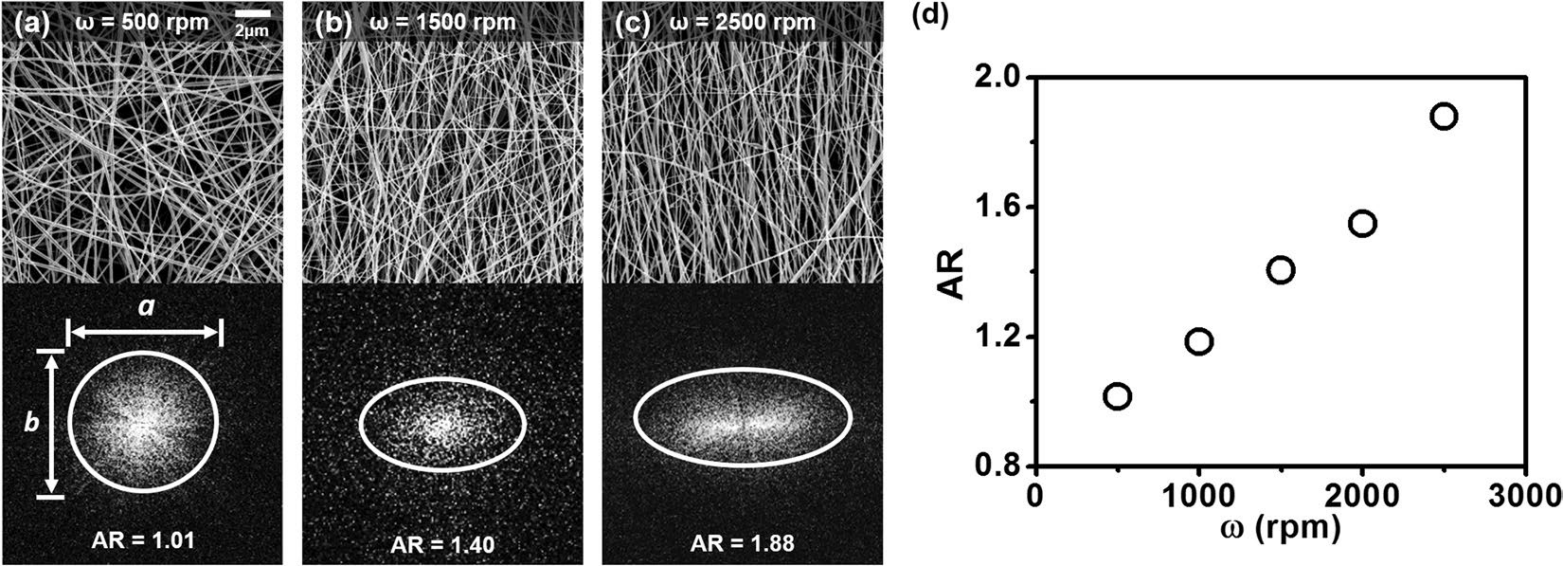
(**a**–**c**) (Top) Representative SEM images of electrospun SF nanofibers assembled at collection drum speeds of (**a**) 500 rpm, (**b**) 1,500 rpm, and (**c**) 2,500 rpm. (Bottom) Amplitudes of the 2D Fourier transforms of (**a**), (**b**), and (**c**). Here, (**a** and **b**) denote the FWHMs of the averaged profiles along the x and y directions, respectively. (**d**) Aspect ratios (ARs) evaluated as a/b for the scaffolds fabricated with different speeds of collection drum.

Figure 1a–c shows representative scanning electron microscope (SEM) images of the SF nanofibrous scaffolds. The fabrication protocol of the SF nanofibrous random laser is illustrated in Methods and Fig. S1. As a gain medium, fluorescent dye molecules, Rhodamine B (RhB), are employed, and scattering structures are formed via electrospinning to control the nanofiber orientation in a continuous manner. It is evident that operating the electrospinning system at various rotational speeds of the collection drum (ω) enables production of the scaffolds with different SF nanofiber arrangements. As the rotational speed of the collection drum increases, the SF nanofibers tend to be well aligned along a certain direction. This behavior may be partly attributed to adhesion and stretching of the fibers on the collection drum during the operation. In other words, at higher ω, the fibers adhered to the surface of the collection drum are stretched, forming aligned arrangement of SF nanofibers. Image analysis using the NIH ImageJ program indicates that the fiber angles from the vertical axis of each SEM image vary from ~±70° at 500 rpm to <±15° at 2,500 rpm. Mean diameter of the SF nanofibers is measured to be 92 ± 20 nm. Shown in the bottom of Fig. 1a–c are the amplitude maps of two-dimensional (2D) Fourier transform of the SEM images. Random nanofiber orientation, or an isotropic distribution in spatial frequency, can be found at small ω, while alignment of SF nanofibers, represented by anisotropic distribution in spatial frequency, can be obtained at larger ω. To represent the SF nanofiber orientation, the aspect ratio of the spatial frequency distribution is evaluated as AR = a/b, where a and b denote the full-width at half-maximum (FWHM) lengths of the spatial frequency distributions along the x and y axes, respectively. Figure 1d shows the ARs of the SF scaffolds as a function of collection drum speeds, which suggests a linear relationship of the ARs with the rotational speed of collection drum.

### Young’s moduli of SF nanofibrous scaffolds

**Figure 2:**
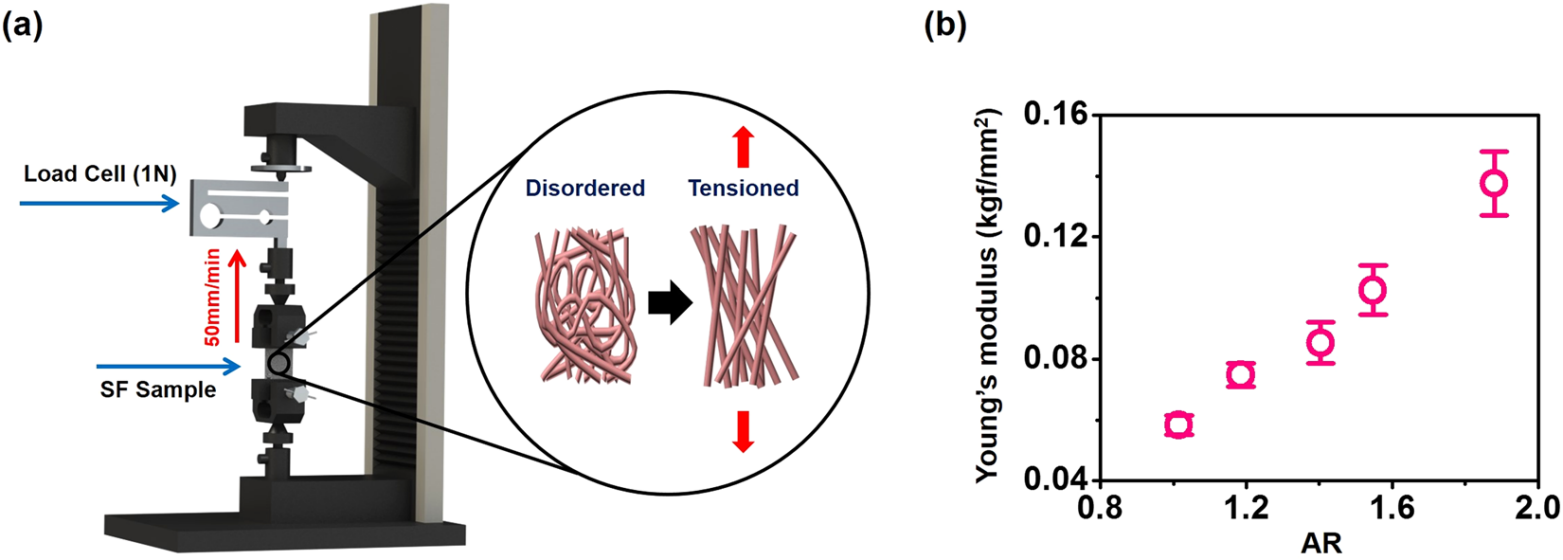
(**a**) An experimental setup to measure Young’s moduli of SF nanofibrous scaffolds. (**b**) Measured Young’s moduli as a function of ARs.

The mechanical properties of the scaffolds were then measured to obtain their dependence on the structural arrangement SF nanofibers (Fig. 2 and Methods). The SF scaffolds were mounted in a Universal Testing Machine (OTT-001, Oriental TM, Republic of Korea), and the tensile tests were performed based on the ASTM D882 standard test method (Fig. 2a). Figure 2b shows the measured Young’s moduli of the SF scaffolds as a function of ARs. A linear behavior of Young’s moduli of the scaffolds with ARs can be observed. The SF scaffolds with aligned nanofibers exhibit higher Young’s moduli. This response can be accounted for as follows: Under a tensile load, individual nanofibers are initially stretched and subsequently carry the tensile load. Therefore, at the same strain, the scaffolds with randomly distributed SF nanofibers are expected to exhibit smaller resistance than those of aligned SF fibers. This is reflected by the larger Young’s moduli of the scaffolds with aligned SF nanofibers.

### Lasing characteristics of SF nanofibrous scaffolds

**Figure 3:**
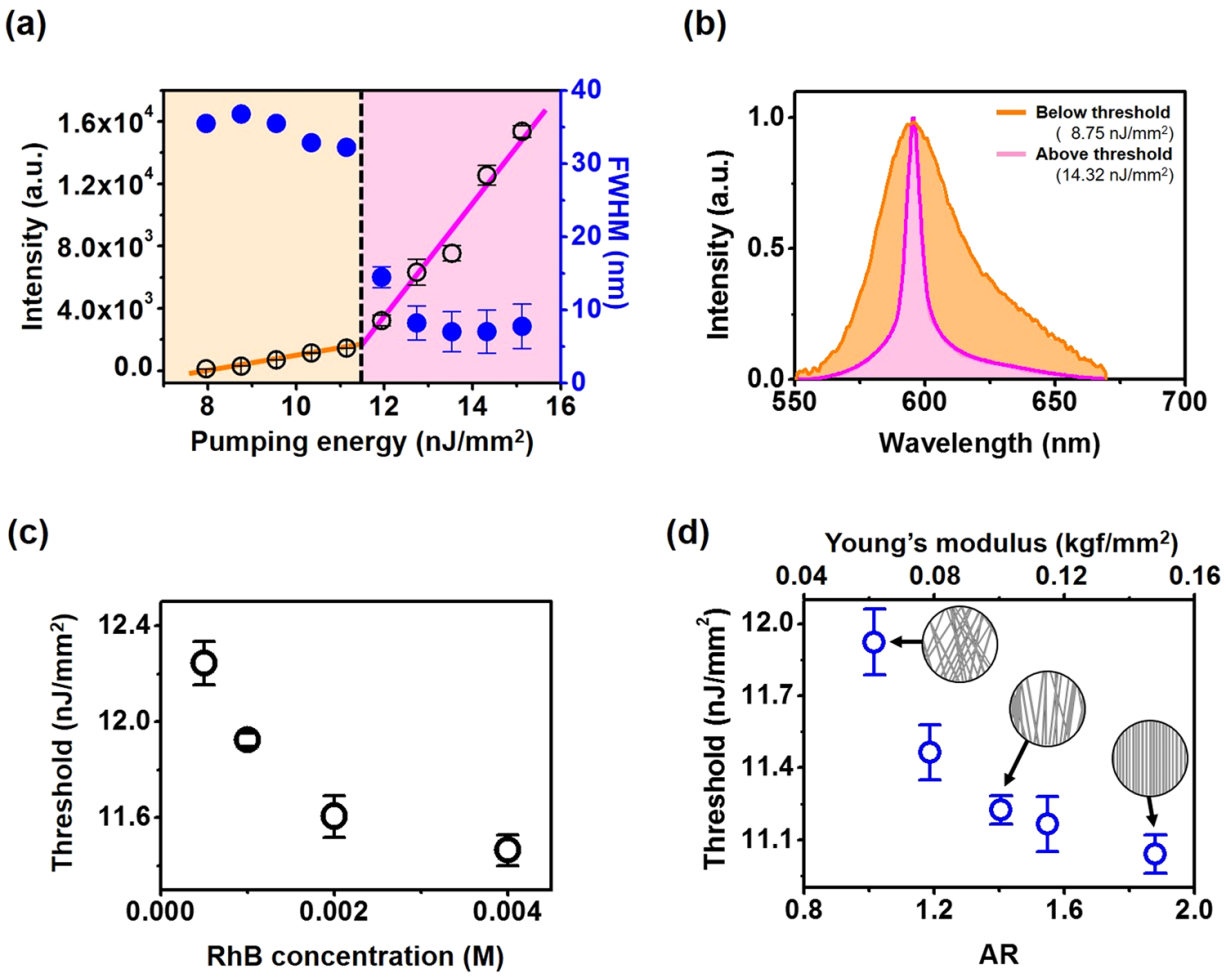
(**a**) Representative intensities (empty black circles), and linewidths (full blue circles) of SF scaffold as a function of pump light energy. Magenta region indicates lasing regime above the threshold (11.46 nJ/mm^2^). These results are obtained with the scaffolds fabricated at a drum speed of 1,000 rpm (AR = 1.18). (**b**) Normalized lasing emission spectra below (orange solid line) and above (magenta solid line) the threshold. (**c**) Measured lasing thresholds as a function of RhB concentrations. (**d**) Lasing thresholds vs. ARs (bottom) and Young’s moduli (top). The RhB concentration was 0.001 M.

We now demonstrate light amplification actions from RhB-doped SF nanofibrous scaffolds (Fig. 3). Details of the photoluminescence (PL) experiment are provided in Methods. Figure 3a presents representative intensities and linewidths of the output emission from a scaffold assembled at the drum speed of 1,000 rpm. The RhB concentration was 0.001 M. The transition of emission regime is marked by both a spectral narrowing and increase of emission power. The threshold is measured to be 11.46 nJ/mm^2^ of pump energy, which compares favorably to similar systems in other materials^42, 43^. Figure 3b shows normalized emission spectra below the threshold at 8.75 nJ/mm^2^ and above the threshold at 14.32 nJ/mm^2^. The lasing emission spectra beyond the threshold exhibits a rather broad single emission peak with a FWHM linewidth of ~8 nm, which are typically observed in diffusive random lasing systems with non-resonant feedback. The lasing thresholds of the scaffold as a function of RhB concentrations are also examined (Fig. 3c). It can be noted that the threshold decreases monotonically, as the RhB concentration increases up to 0.004 M. This behavior may be explained by a larger number of gain molecules at higher RhB concentrations, and is consistent with the experimental results in other publications^44, 45^. Comparisons of the measured lasing thresholds against the degrees of SF nanofiber alignment (i.e., AR) and Young’s moduli are shown in Fig. 3d. For this experiment, the RhB concentration in the scaffolds was set to 0.001 M. The lasing threshold decreases, as the nanofibers are aligned into parallel arrays normal to the pump beam direction. It can also be seen that the lasing thresholds vary inversely with the Young’s modulus of the scaffolds. This result may be of significance in some applications, as it implies that alteration in the structural arrangement and mechanical properties of the scaffolds can be probed by measuring the lasing signatures (e.g., lasing threshold).

### Numerical validation

**Figure 4:**
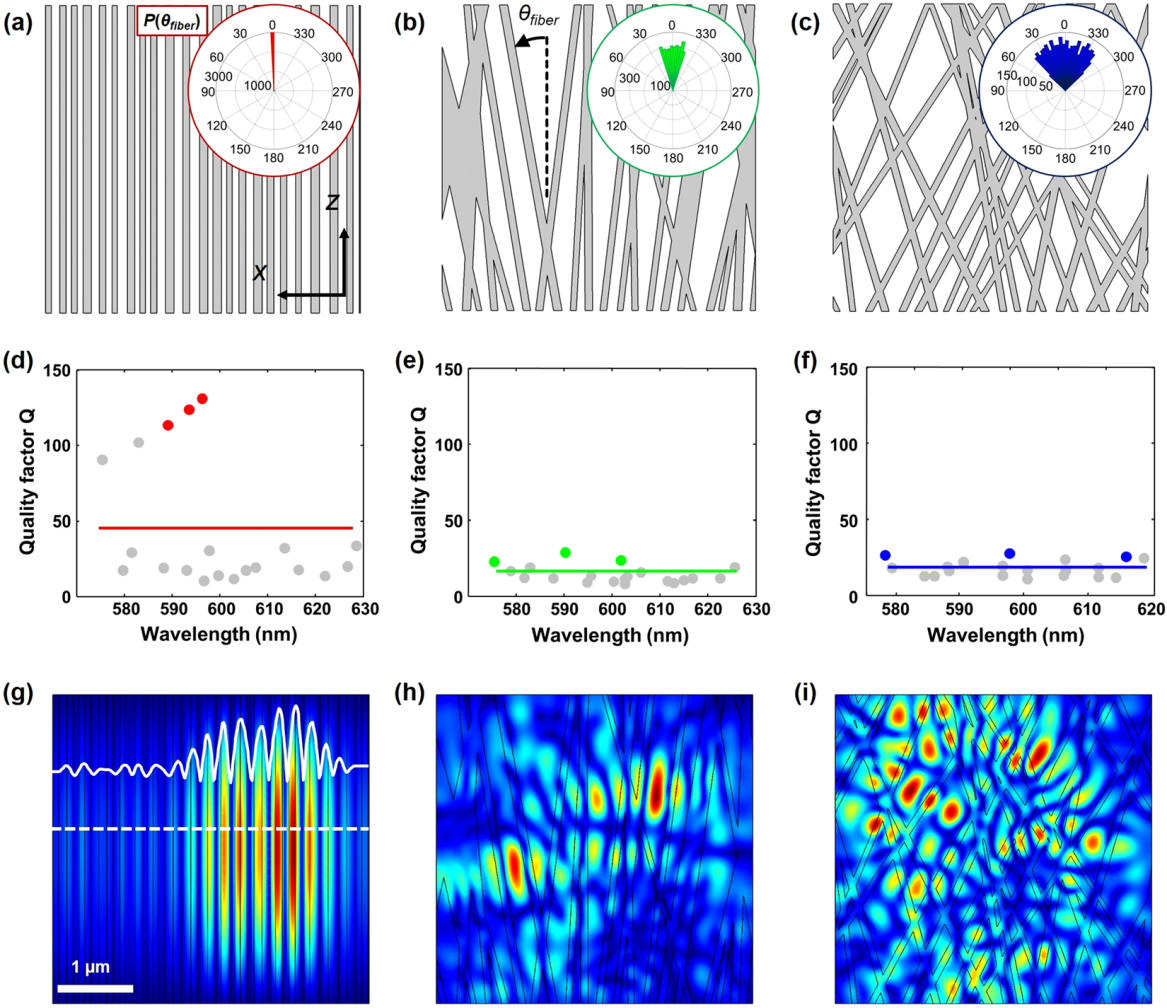
(**a**–**c**) SF nanofiber configurations for the FEM quasi-mode analysis. Histograms of the angular distribution of the SF nanofibers are presented in the insets of (**a**–**c**). (**d**–**f**) Quality factors as a function of wavelengths for the three cases. Solid lines denote the *Q*_*AVE*_. (**g**–**i**) Amplitudes and spatial distributions of the electric fields corresponding to the three modes with the highest *Q* factors. These modes are indicated by the red, green and blue dots in (**d**–**f**), respectively.

To further validate the observed lasing behaviors in the SF scaffolds of diverse nanofiber arrangements, the quasi-modes in the scaffolds are examined through the FEM analysis in COMSOL (Fig. 4). Note that lasing modes could be different from the quasi-modes for a weakly scattering regime, but are still associated with individual quasi-modes of the passive system. Figure 4a–c shows the spatial distribution of SF nanofibers, along with the histograms of their angular distributions that have been used in the analysis. Quality (*Q*) factors for the quasi modes are obtained (grey circles in Fig. 4d–f), and the average *Q* factors are also evaluated as a representative parameter for the lasing characteristics in each case (solid lines in Fig. 4d–f). The average *Q* factors are defined as ${Q}_{AVE}=|\sum _{m=1}^{M}Re(\kappa )/({M}^{2}\sum _{m=1}^{M}2Im(\kappa ))|$, where *κ* represents the complex eigenvalue of eigenmode in the lasing system, *m* is the mode number, and *M* is the total number of modes. As the SF nanofibers are aligned reducing the structural dimensionality, the *Q* factors become larger, which corresponds to smaller loss in the lasing system. These results are consistent with the experimental observations in Fig. 3d, where smaller lasing thresholds are measured in the aligned SF nanofibers. Figure 4g–i shows the spatial distribution of the three representative electric fields with the highest *Q* factors for each SF scaffold (colored circles in Fig. 4d–f). In large angular variations (Fig. 4i), the waves scatter into all different directions, but in small angular variations of nanofibers (Fig. 4g), the volume explored by the waves is restricted to the direction perpendicular to the fiber direction. These results explain the measured lasing characteristics in the SF nanofibers of different arrangements.

### Measurement of lasing signatures from SF nanofibers under tensile loading

**Figure 5:**
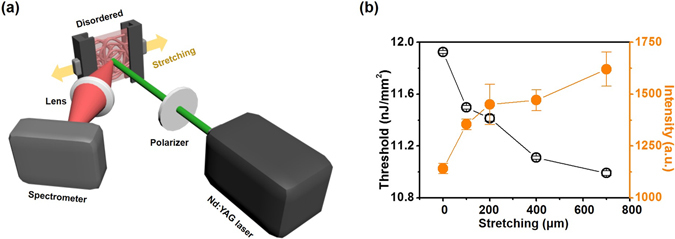
(**a**) PL measurement setup combined with tensile setup (**b**) Measured thresholds and output emission intensities at various tensile loads. The output emission was measured at a pump energy of 12 nJ/mm^2^.

The measured interrelationships among optical-mechanical-structural properties of the SF scaffolds (Fig. 3d) can be utilized as a transduction mechanism for non-contact *in-situ* optical sensor of mechanical and structural properties. In order to demonstrate its utility, we performed measurement of lasing features from a SF nanofibrous scaffold, while the scaffold is under tensional loads. The two opposite sides of the scaffold assembled at 500 rpm were clamped to separate translational stages to stretch the scaffold. (Fig. 5a and Methods). The scaffold was then incrementally stretched, and its lasing properties were measured. Shown as empty black circles in Fig. 5b are the measured lasing thresholds at increasing tensile loads. As the scaffold is stretched, the lasing thresholds were found to decrease. This behavior can be explained by the observation made in Fig. 3d. As the scaffold is stretched, the SF nanofibers are likely to be more aligned, making a transition from small to larger ARs. As the AR increases, the scaffolds are expected to exhibit smaller lasing thresholds. The full orange circles in Fig. 5b denote the optical power of output emission measured at a pump energy beyond the lasing threshold (12 nJ/mm^2^). The optical power of the output emission from the scaffold increased as the SF nanofibers are stretched further with higher loads. This can also be accounted for by the measured smaller lasing threshold for the aligned SF nanofibers. Interestingly, the emission wavelength from the scaffold did not vary under various tensile loads (data not shown). This experimental result suggests a potential utility of this lasing system for monitoring alteration of local mechanical arrangement and properties.

## Discussion

Our experimental and calculation results can be adequately described by diffusive models^46^. Under the illumination of a pump laser, the excited molecules emit photons through spontaneous emission after a given time. The emitted photons travel along random paths, and either generate stimulated emission if they encounter excited molecules, or are absorbed. The initial spontaneous emission will be amplified so long as it travels in the excited region. For the pumping beam normal to the direction of the fiber alignment, the highly forward and backward scattering redirect the emitted photons to the excited volume with high probability. Therefore, an enhanced population inversion and a smaller lasing threshold could be obtained in the SF scaffold with aligned fibers. For the scaffolds with randomly distributed SF fibers, random light scattering results in less confinement of the emitted photons in the excited region. This explains the smaller *Q* factors and larger lasing threshold in the random SF scaffolds, as demonstrated in our FEM analysis. In order to validate the enhanced directional scattering and emission in the aligned SF nanofibrous scaffolds, we measured angular distributions of output emissions from the scaffolds assembled at various collection drum speeds (Methods and Fig. S2). It is clearly seen from Fig. S2 that the output emissions from the aligned SF nanofibers exhibit directional emission normal to the direction of the SF nanofiber alignment, while the emission from the random SF nanofibers is characterized by a broad angular distribution. This observation clearly accounts for the enhanced directional scattering and emission processes in the aligned SF nanofibers. We then examined operating regime of SF nanofibrous random lasers by measuring transport mean free path-lengths (*l*_*t*_) of the fabricated SF scaffolds via a coherent back-scattering setup (Fig. S3 and Methods). The measured *l*_*t*_ of the scaffolds were in the range 12 ~ 15.4 μm, which corresponds to 20 times the emission wavelength. Dimensionless parameter *kl*_*t*_ were found to be in the range of 119 ~ 153, where *k* is the wave number of light. It implies that the scaffolds are scattering materials well described by diffusive models^46^.

SF nanofibrous structure has previously been explored as the scaffolds for cell proliferation and drug delivery^47, 48^. It exhibits high surface to volume ratio, and can therefore interact with host biological systems or external environments with high contact areas. On the other hand, random arrangement of the SF nanofibers provides a great platform for light amplification, but lasing from these structures and its dependence on structural and mechanical features have not been studied thus far. Random lasing from other fibrous structures has been reported^49, 50^, but its features were examined in other perspectives, for example, in the context of plasmonic enhancement and formation of random resonators^42, 43, 51^. To demonstrate light amplification in SF nanofibers, we incorporated rhodamine B dyes into the silk fibroin and assembled random structures as modulating directionality and dimensionality through an electrospinning method. Our results demonstrate that light amplification or random lasing can be engineered through the control of structural arrangement or dimensionality via electrospinning. Lasing features, structures and mechanical properties of the fabricated SF scaffolds were then examined to obtain their interrelationships.

We envision that the SF nanofibrous random laser is promising for sensing applications, as it is flexible, largely insensitive to external shape and versatile to host diverse gain materials. The scaffold can also be readily tuned to have certain mechanical properties through the controlled arrangement of SF nanofibers. Integration of such device in biological tissues and measurement of its lasing features based on the observed interconnects with structural and mechanical properties may allow non-contact measurement of the structural modifications of the scaffolds in the host systems. SF nanofibers have been structured to form composites with controlled mechanical strengths by incorporating various nanoparticles^52, 53^. For instances, hydroxyapatite^54, 55^ and chitosan^56, 57^ nanoparticles have been employed to tune the mechanical properties of the SF-based composites. Once the composite materials are integrated to biological systems, it then becomes critical to measure degradation or alteration of their mechanical properties in the host tissues in continuous and non-destructive manner. Measuring lasing features in the composites could be a feasible means to non-destructive, non-contact monitoring of their mechanical properties. SF-based nanofibrous scaffolds have also been explored in tissue engineering^58–61^. In particular, scaffolds made of aligned nanofibers have been regarded as favorable ones for nerve regeneration and directed cell growth due to their superior cell attachment and proliferation^62–64^. The structural modification of the SF-based scaffolds due to the cell infiltration, growth and proliferation may be associated with changes in their mechanical and optical properties, and so could be monitored by examining their lasing features. We also note that the spatial resolution of the sensor can be varied depending on the optical beam size. Therefore, two-dimensional mapping of structural and mechanical alterations can be potentially achieved by scanning the optical beam across the SF scaffolds. The incorporation of clinically viable dyes (e.g., indocyanine green and methylene blue) into the SF scaffolds may also open up novel directions towards its applications to *in vivo* studies.

In summary, we have demonstrated light amplification in RhB-doped SF nanofibers. By operating the electrospinning system at various rotational speeds of collection drum, we have controlled the alignment of SF nanofibers in structuring the scaffolds. The PL results indicate that the scaffolds act as a diffusive random lasing system with its lasing features strongly related to structural and mechanical properties of the scaffolds. These lasers may therefore represent opportunities in the context of sensor devices since the local perturbation of mechanical properties could lead to alterations in the lasing features. The random lasing systems based on SF nanofibers are compatible with living tissues due to biocompatible constituents, and may also be intact with biological systems with high surface to volume ratios. All these features suggest its potential as a biocompatible tool to sense or image local mechanical and structural properties with high sensitivity.

## Methods

### Fabrication of silk fibroin scaffolds

*Bombyx mori* cocoon was prepared for a SF solution based on the protocols described^65^. In brief, *Bombyx mori* cocoon was degummed to remove sericin. The degummed silk was then dissolved in a 9.3 M LiBr (SAMCHUN Chemical, South Korea, L1106) solution and dialyzed against deionized (DI) water for 3 days. After dialysis, the SF/DI solution was freeze-dried and dissolved in 98% formic acid (JUNSEI Chemical Co, Japan, 25010-0350) at a concentration of 10% (w/v). SF scaffolds were then fabricated via electrospinning, which provides rapid and low-cost strategy to control the SF nanofiber alignment in a continuous manner. As a gain medium, rhodamine B (Sigma, St. Louis, MO, USA, 1001405226) was added to the SF solution. Concentration of the rhodamine B was 0.001 M, unless specified otherwise. The electrospinning system (NanoNC, South Korea, ESR200RD) consisted of a high-voltage power supply, syringe pump, metal nozzle, and rotating collection drum. The alignment of the SF nanofibers was achieved by controlling the rotational speed of the collection drum. The rotational speed of the drum was varied from 500 to 2,500 rpm. The SF solution was continuously fed to the tip of the charged metal nozzle via the syringe pump at a constant speed. An electric field of 1.2 ~ 2.0 kV/cm was applied between the nozzle and the collection drum.

### Tensile test setup

The experiment was performed with a Universal Testing Machine (OTT-001, Oriental TM, Republic of Korea), based on the ASTM D882 standard test method. Tensile testing was performed at a speed of 50 mm/min at room temperature with a load cell capacity of 1 N. The SF specimen had a width of 30 mm and a length of 30 mm.

### PL experiment

A 532 nm Q-switched mode-locked Nd:YAG laser (LOTIS TII Inc., LS-2131) was employed as a pumping light source. The repetition rate and width of the laser pulse were 10 Hz and 10 ns, respectively. The output beam from the pump laser was delivered to the SF scaffold with a diameter of ~4 mm. The optical energy of the pumping laser was controlled by a polarizer (Thorlabs Inc., LPVISE100-A), and increased from 90 to 190 nJ in steps of 10 nJ for each PL measurement. The output emission from the scaffold was collected via a lens with a focal length of 30 mm (Thorlabs Inc., AC254-030-A-ML) and directed to a spectrometer with a spectral resolution of ~0.1 nm (Ocean Optics Inc., customized HR4000). The integration time of the spectrometer was set to ~95 ms.

Once the output emission from a SF nanofibrous scaffold is measured as a function of optical pump energy, the lasing threshold was determined at the intersection of the linear fits performed in the regions of the spontaneous and stimulated emissions in the light input–output plot. We repeated the threshold measurements of a SF scaffold assembled at 1000 rpm over 5 times, and found that the measurement precision was found to be 0.26 nJ/mm^2^ in standard deviation. For all of the experiments, the scaffold thickness was >300 μm.

### FEM calculation

The diameters and angular orientations of SF nanofibers were obtained from the representative SEM images (Fig. 1a–c); three SF scaffolds with different orientations of nanofibers (*θ*_*fiber*_) were considered. The refractive indices of the nanofibers and air were set at 1.54 and 1, respectively. The geometrical distributions were applied to the computational domain of the FEM of 40 µm × 40 µm, which made it possible to obtain a numerical solution of the two-dimensional Helmholtz equation. 2D Helmholtz equation was discretized and formulated into the generalized eigenvalue problem, and *E*_*z*_, *H*_*x*_, and *H*_*y*_ components of the transverse magnetic (TM) mode are considered, in which the electric field is along the cylindrical axis, in a manner similar to a previous report^66^. For the boundary conditions, a layer perfectly matched to the surrounding medium of the scattering structures was employed. By the integrating boundary conditions, the numerical solutions of the generalized eigenvalue problem were obtained by an unsymmetric-pattern multifrontal method for sparse lower and upper triangular matrices (LU) factorization. Instead of the field intensity, the norm of *E*_*z*_ was visualized for a better display of the small values of the field. We performed FEM analysis with the RF Module of COMSOL Multiphysics (version 4.3a), and examined 20 leaky modes around λ = 600 nm. For simplicity, the effect of the magnetic field on the scaffolds was considered to be negligible (i.e., magnetic permeability = 0).

### PL measurement with SF nanofibers under tensile loads

A 30 mm × 30 mm SF scaffold assembled at a collection drum speed of 500 rpm (AR = 1.01) was used. The two opposite sides of the specimen were clamped by holders (Edmund Optics inc., #54-994), that were mounted onto mechanical translation stages (Newport Co., Ltd., M-423-MIC). The specimen was stretched from 0 μm to 700 μm. PL measurements were performed as described above for each displacement of the stages.

### Angular distribution of output emissions from SF nanofibers

The experimental PL setup is illustrated in Fig. S2a. The setup is similar to the PL setup as described above, except that the spectrometer was mounted on a rotational arm (Thorlabs Inc., LC1A) to measure output emissions from SF scaffolds at various detection angles. The spectrometer was elevated by 30 degrees in order not to block the beam path of the pump laser. For measurement, detection angle (θ) was varied from −85 to 85 degrees in steps of 10 degrees, and the pumping energy was set to 13.84 nJ/mm^2^, which was above the measured threshold (11.04~11.92 nJ/mm^2^). The distance from the scaffolds to the spectrometer was set to 70 mm. The SF scaffolds were assembled at a drum speed of 500, 1000, 1500, and 2500 rpm, producing the nanofiber arrangements with the aspect ratios (ARs) of 1.01, 1.18, 1.40 and 1.88. The concentration of rhodamine B in the scaffolds was 0.001 M.

### Coherence backscattering (CBS)

To quantify the bulk (passive) optical properties of the fabricated SF scaffolds, we employed a coherent backscattering (CBS) method to measure the *l*_*t*_ of light. The CBS setup was similar to that of ref. 67, except that the experiment was performed with a 633 nm laser (Edmund Optics Inc., Model 1135 P). Its wavelength is close to the emission wavelength of RhB dye (~592 nm). Dye-free scaffolds were utilized for the experiment to avoid light absorption. The frame rate of the employed image sensor (IO Industries Inc., 4M180MCL) was 10 fps. The measurement was performed for 10 s, while the specimen was jittered continuously during the measurements, which removed strong speckle patterns in the angular signals. The measured CBS cone was fitted with θ ≈ λ/(3π*l*_*t*_), and *l*_*t*_ of each sample was extracted from the fit^68^. Here, θ is a full width at half-maximum of the CBS cone and λ is the wavelength of light.

## Electronic supplementary material

Supplementary Information
