# Supplementary material for: Random lasing from structurally-modulated silk fibroin nanofibers
Source: Sci Rep. 2017 Jul 3;7:4506. doi: 10.1038/s41598-017-04881-5 (PMC5495807; doi:10.1038/s41598-017-04881-5)
Supplement: Supplementary file 1 — Supplementary Information [file 41598_2017_4881_MOESM1_ESM.pdf]

Random lasing from structurally-modulated silk fibroin nanofibers

Authors: Soocheol Kim[1,†], SungYeun Yang[1,†], Seung Ho Choi[2], Young L. Kim[2], WonHyoung Ryu[1,*] & Chulmin Joo[1,*]

e-mail: whryu@yonsei.ac.kr (WonHyoung Ryu), cjoo@yonsei.ac.kr (Chulmin Joo)

[1] School of Mechanical Engineering, Yonsei University, Seoul, 03722, Republic of Korea

[2] Weldon School of Biomedical Engineering, Purdue University, West Lafayette, Indiana 47907, USA

[†] These authors contributed equally to this work

* These are corresponding authors

Fabrication protocol of silk fibroin nanofibrous random laser

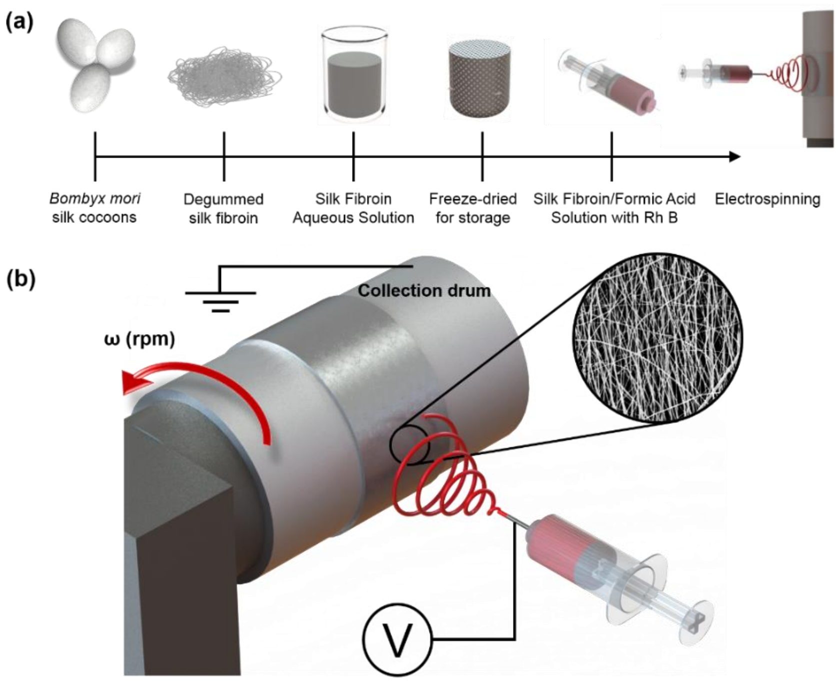

Figure S1. (a) Production of biocompatible random lasers composed of electrospun RhB-SF nanofibers. (b) Control of SF nanofiber alignment is achieved by operating the electrospinning system at various rotational speed of collection drum (ω).

Angular distribution of emission from SF nanofibrous random lasers

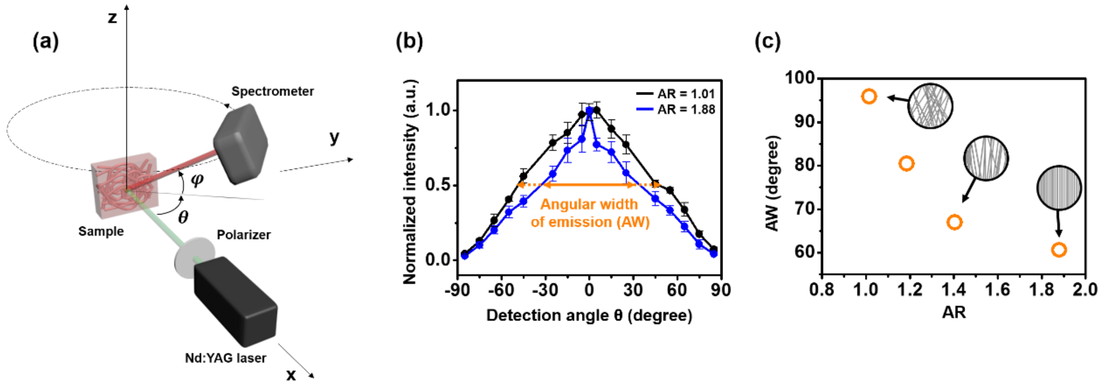

Figure S2. (a) Experimental setup for measuring angular distribution of output emission from SF nanofibrous scaffolds. The scaffold was on the y-z plane, and the pump laser (i.e., Nd:YAG laser) illuminated the scaffold along the x axis. The spectrometer, mounted in a rotational arm, was elevated by $\varphi = 30°$ in order not to block the beam path of the pump laser. The θ denotes the detection angle. (b) Representative angular emissions from the SF scaffolds with AR = 1.01 (black line) and AR = 1.88 (blue line). The angular emission for each scaffold was normalized by its maximum emission at $\theta = 0°$. The aligned SF nanofibers (AR = 1.88) exhibited a sharp output emission around $\theta = 0°$, while the SF scaffold with a random fiber arrangement (AR = 1.01) was characterized by a broad angular distribution. The errorbar denotes the standard error evaluated over five measurements. (c) Angular width (AW) of output emissions from the SF nanofibers with different structural arrangements. The angular width of emission for each scaffold was determined as the full-width at half-maximum of its angular output emission. It can be seen that the aligned SF nanofibers exhibit a narrower angular width of emission, indicating enhanced directional scattering and emission in the scaffolds.

Transport mean free path-lengths of SF scaffolds

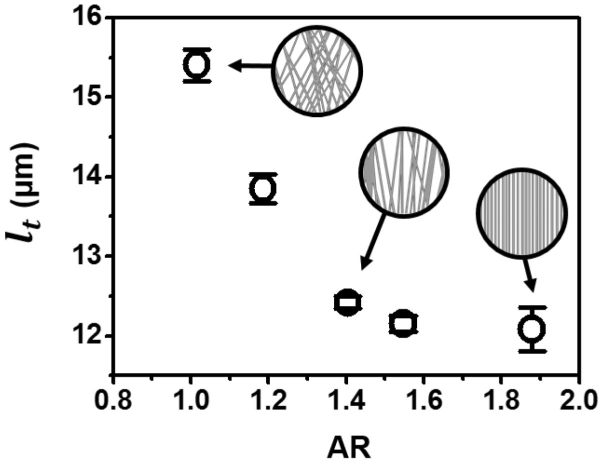

Figure S3. Transport mean free paths as a function of ARs. The errorbar denotes the standard error evaluated over five measurements.
